# Elesclomol: a copper ionophore targeting mitochondrial metabolism for cancer therapy

**DOI:** 10.1186/s13046-022-02485-0

**Published:** 2022-09-12

**Authors:** Peijie Zheng, Chuntao Zhou, Liuyi Lu, Bin Liu, Yuemin Ding

**Affiliations:** 1 Department of Clinical Medicine, School of Medicine, Zhejiang University City College, Hangzhou, 310015 China; 2 Institute of Translational Medicine, Zhejiang University City College, Hangzhou, 310015 China; 3 Key Laboratory of Novel Targets and Drug Study for Neural Repair of Zhejiang Province, Zhejiang University City College, Hangzhou, 310015 China

**Keywords:** Elesclomol, Cuproptosis, Mitochondrial metabolism, Cancer, Anticancer drugs, Cancer stem cells, Drug safety, Clinical trials

## Abstract

Elesclomol is an anticancer drug that targets mitochondrial metabolism. In the past, elesclomol was recognized as an inducer of oxidative stress, but now it has also been found to suppress cancer by inducing cuproptosis. Elesclomol’s anticancer activity is determined by the dependence of cancer on mitochondrial metabolism. The mitochondrial metabolism of cancer stem cells, cancer cells resistant to platinum drugs, proteasome inhibitors, molecularly targeted drugs, and cancer cells with inhibited glycolysis was significantly enhanced. Elesclomol exhibited tremendous toxicity to all three kinds of cells. Elesclomol's toxicity to cells is highly dependent on its transport of extracellular copper ions, a process involved in cuproptosis. The discovery of cuproptosis has perfected the specific cancer suppressor mechanism of elesclomol. For some time, elesclomol failed to yield favorable results in oncology clinical trials, but its safety in clinical application was confirmed. Research progress on the relationship between elesclomol, mitochondrial metabolism and cuproptosis provides a possibility to explore the reapplication of elesclomol in the clinic. New clinical trials should selectively target cancer types with high mitochondrial metabolism and attempt to combine elesclomol with platinum, proteasome inhibitors, molecularly targeted drugs, or glycolysis inhibitors. Herein, the particular anticancer mechanism of elesclomol and its relationship with mitochondrial metabolism and cuproptosis will be presented, which may shed light on the better application of elesclomol in clinical tumor treatment.

## Background

In the past, aerobic glycolysis was thought to be the primary energy source for cancer cells because many cancers showed enhanced glycolysis even when oxygen was abundant [1]. This phenomenon is known as the Warburg effect [1]. However, increasing evidence challenges this view since mitochondrial metabolism has also been found to be a crucial energy source for some cancer cells [2]. The spontaneous enhancement of mitochondrial metabolism has been found in various cancers, including melanoma, breast cancer and leukemia [2]. Additionally, the survival of drug-resistant cancer cells and cancer stem cells is also heavily reliant on energy provided by mitochondrial respiration [2, 3]. In fact, mitochondrial metabolism plays a critical role in tumorigenesis, proliferation, metastasis, and drug resistance [4]. Therefore, targeting mitochondrial metabolism is an effective strategy for cancer suppression.

Inhibitors of oxidative phosphorylation (OXPHOS) have been shown to be effective cancer suppressors that rely on mitochondrial metabolism [3]. However, OXPHOS inhibitors may also inhibit immune cells with the same metabolic profile as cancer cells [2]. Considering the critical role of immune mechanisms in the body's anticancer efforts, the blind use of mitochondrial respiration-inhibiting drugs may be counterproductive [5, 6]. Moreover, some classical mitochondrial toxic drugs lead to unacceptable side effects, which are also problematic in targeting mitochondrial metabolism for cancer therapy [3]. We noticed a small molecule compound called elesclomol, which is an anticancer drug that induces oxidative stress in cancer cells, and that enhanced mitochondrial metabolism makes cancer cells sensitive to elesclomol [7]. Given that several clinical trials have validated the safety of elesclomol and the cytotoxicity of elesclomol selectively targets cancer cells [6], it is expected to be a safe and effective anticancer agent. It is worth noting that eleslcomol did not induce significant reduction in basal or adenosine triphosphate (ATP)-linked respiration by targeting the electron transport chain (ETC) directly, but rather significantly reduced the spare capacity of respiration by inhibiting components of the tricarboxylic acid (TCA) cycle [7].

Elesclomol is a chemotherapeutic adjuvant developed by Synta Pharmaceuticals and originally developed for treating metastatic melanoma [8]. In subsequent clinical trials, a sodium salt formulation of elesclomol was developed for use in combination with paclitaxel or alone for treating multiple solid tumors and acute myeloid leukemia [5, 9–11] (Table 1). Published data show a favorable safety profile for elesclomol alone or in combination with paclitaxel, but patients are less sensitive to elesclomol. Except for a small phase II trial in patients with stage IV melanoma, which found that elesclomol was effective in prolonging progression-free survival (PFS), elesclomol did not produce a desired clinical response [5, 9–11]. While a subsequent phase III trial demonstrated that elesclomol combined with paclitaxel did not prolong PFS, this study found that patients with low lactate dehydrogenase (LDH) levels may be more sensitive to elesclomol [12]. The elevated serum LDH level in tumor is usually related to hypoxia, which forces tumor cells to supply energy through enhanced glycolysis [13]. This also strongly hints at the relationship between cancer sensitivity to elesclomol and cellular metabolism. In the last two years, there has been renewed interest in elesclomol [14], whose specific transport function of copper ions to cellular mitochondria suggests its potential therapeutic for rare diseases of copper deficiency, such as the Menkes disease [15]. Here, we propose that the prospect of elesclomol in cancer metabolic therapy deserves special attention.

**Table 1:** Elesclomol-related clinical trials

Clinical Trial Subjects	Staging of Clinical Trials	Recruitment Status	Drug use	Results	References
Acute myeloid leukemia	Phase I	Unknown	Elesclomol sodium	Elesclomol has a good clinical safety profile, but patients have not developed clinical responses to elesclomol	26732437
Solid tumors	Phase I	Completed	Elesclomol sodium, Paclitaxel	The combination of Elesclomol and paclitaxel was well tolerated by patients and the toxicity profile of elesclomol was similar to that of single agent paclitaxel	17255281
Melanoma	Phase I/II	Completed	Elesclomol sodium, Paclitaxel	The combination of elesclomol and paclitaxel resulted in a statistically significant doubling of median PFS, with an acceptable toxicity profile and encouraging OS	19826135
Melanoma	Phase III	Terminated	Elesclomol sodium, Paclitaxel	The combination of elesclomol and paclitaxel did not significantly improve PFS	23401447
Recurrent or persistent ovarian epithelial cancer, fallopian tube cancer, primary peritoneal cancer	Phase II	Completed	Elesclomol sodium, Paclitaxel	The combination of elesclomol and paclitaxel was well tolerated by patients but the proportion responding was low	30309721
Solid tumors	Phase I	Suspended	Elesclomol sodium	Unpublished	-
Prostate cancer	Phase I	Completed	Elesclomol sodium, Docetaxel	Unpublished	-
Stage IIIB/IV non-small cell lung cancer	Phase I/II	Completed	Elesclomol sodiuml, Paclitaxel, Carboplatin	Unpublished	-
Soft tissue sarcoma	Phase II	Completed	Elesclomol sodium, taxane	Elesclomol enhanced taxane efficacy by induction of Hsp70	16784029

Elesclomol-related clinical trials were searched using the ClinicalTrials.gov platform. Nearly a dozen clinical trials related to elesclomol treatment have been conducted with the estimated enrollment of a thousand patients with different types of cancers, including melanoma, ovarian cancer, and acute myeloid leukemia. Unfortunately, according to published results, the clinical anticancer efficacy of elesclomol is suboptimal, but patients have good tolerance to elesclomol

*PFS* Progression-free survival, *OS* Overall survival

The anticancer mechanism of elesclomol has long been interpreted as promoting the accumulation of intracellular reactive oxygen species and ultimately inducing oxidative stress, which involves multiple molecular targets that range from mitochondria to nuclear DNA [6, 16–24]. Meanwhile, the anticancer effect of elesclomol has been found to rely on its ability to transport extracellular copper ions [6, 7] as well as the mitochondrial metabolic intensity of cancer cells [17, 20, 24–29], but a specific mechanism to explain this phenomenon is lacking. Recently, cuproptosis has been proposed as a novel form of cell death, and soon after, elesclomol was found to be a cuproptosis inducer [7, 28]. Excitingly, the occurrence of cuproptosis is also related to mitochondrial metabolism, and the proposed concept of cuproptosis emerged as a distinct mechanism to explain the cancer suppressive effect of elesclomol [7]. The association between elesclomol and cuproptosis also enables people to re-explore the clinical anticancer potential of elesclomol. This review will emphasize the anticancer mechanism of elesclomol and the clinical application value of elesclomol via its targeting of mitochondrial metabolism.

## Mitochondrial metabolism affects the sensitivity of cancer cells to elesclomol

**Fig. 1:**
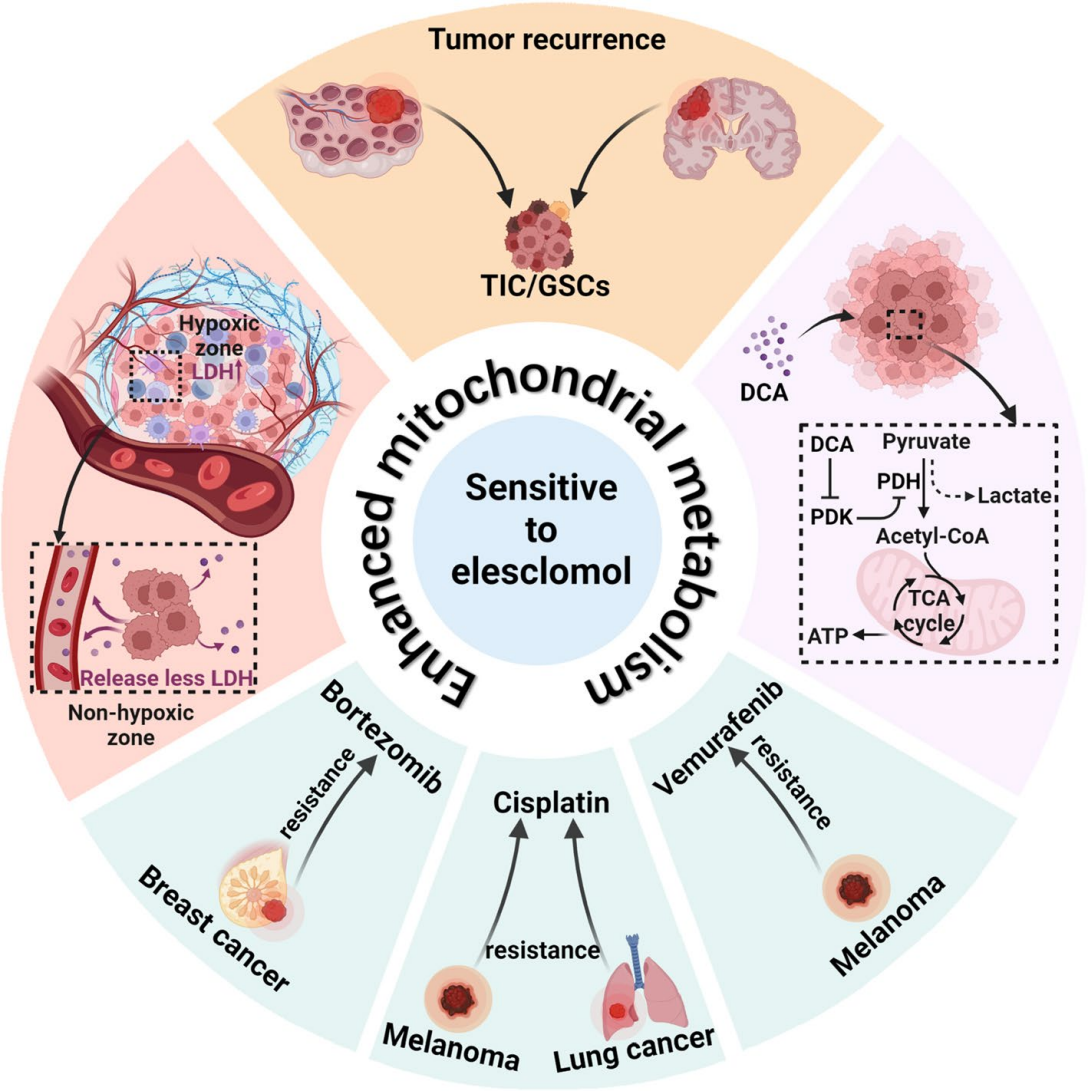
Enhanced mitochondrial metabolism sensitizes cancer cells to elesclomol. Cancer cells highly dependent on mitochondrial metabolism are sensitive to elesclomol, including cancer stem cells, drug-resistant cancer cells, and glycolysis-inhibiting cancer cells. Both TICs in ovarian cancer and GSCs in glioblastoma are associated with cancer recurrence and are highly dependent on mitochondrial metabolism. Drug-resistant cancer cells, including bortezomib-resistant breast cancer cells, cisplatin-resistant melanocytes and lung cancer cells, and vemurafenib-resistant melanocytes, increase their dependence on mitochondrial metabolism in the development of drug resistance. PDK inhibitor DCA enhances mitochondrial metabolism in melanoma cells by shifting their metabolism from glycolysis to oxidative phosphorylation. In addition, hypoxia in solid tumors reduces the intensity of mitochondrial metabolism, and the degree of tumor hypoxia is positively correlated with the serum LDH levels of the patients. Patients with low serum LDH levels are sensitive to elesclomol. ATP: adenosine triphosphate; DCA: dichloroacetate; GSCs: glioblastoma stem cells; LDH: lactate dehydrogenase; PDH: pyruvate dehydrogenase; PDK: pyruvate dehydrogenase kinases; TICs: tumor-initiating cells

The anticancer activity of elesclomol is related to the mitochondrial metabolic state of cancer cells. The higher the reliance on mitochondrial metabolism is, the higher the sensitivity of cancer cells to elesclomol (Fig. 1).

### Cancer stem cells are sensitive to elesclomol

The stem-like tumor-initiating cells (TICs) in ovarian cancer (OC) are associated with the recurrence of ovarian cancer after chemotherapy, which ultimately leads to an abysmal prognosis in OC patients [30]. Using high-throughput drug screening, researchers tested the sensitivity of OC cells cultured under TIC-enriched conditions [31] to 1,978 compounds, of which elesclomol proved to be one of the potent anticancer compounds [29]. Under TIC-enriched conditions, elesclomol treatment reduced the sphere formation [29] of OC cells, a phenotype favorable to TICs [32]. OC cells with a high expression of CD133 and high aldehyde dehydrogenase (ALDH), which are markers of TICs [33], were also reduced [29]. Notably, disulfiram (DSF), another copper ionophore, was also screened as an effective drug [29]. At the same time, OC TICs tended to supply energy by increasing mitochondrial respiration, which may explain their sensitivity to copper ionophores, elesclomol, or DSF.

In addition, some studies have reported that glioblastoma stem cells (GSCs) are also heavily reliant on mitochondrial respiration for energy supply [34]. A drug screen for GSCs revealed that elesclomol at submicromolar concentrations was extremely potent in killing GSCs [24]. Elesclomol treatment resulted in an increase in the mitochondrial membrane potential of GSCs accompanied by an increase in reactive oxygen species (ROS) [24], which are mainly concentrated in mitochondria rather than the cytoplasm, suggesting that mitochondria are potential targets of elesclomol [24].

### Drug-resistant cancer cells are sensitive to elesclomol

#### Platinum-resistant cancer cells

Platinum is currently the first-line drug for clinical cancer treatment, but platinum resistance in cancer cells has greatly limited its clinical application [35]. Drug resistance inevitably occurs in the advanced treatment of melanoma patients, especially when traditional platinum is used [36]. A series of studies has shown that the activation of mitochondrial metabolism is closely related to the development of cisplatin resistance in melanoma [37]. In recent years, it has also been reported that the development of cisplatin resistance in lung cancer cells is associated with metabolic reprogramming, which involves the transformation of glycolysis metabolism to mitochondrial metabolism [26, 38].

Proteomic analysis has identified a group of slow-cycling cells [39, 40] in melanoma that rely on mitochondrial respiration and whose replenishment of the tumor underlies the development of drug resistance and tumor maintenance [27]. This group of cells is characterized by the high expression of Jumonji AT-rich interactive domain 1B (JARID1B) [27]. When melanoma cells were treated with elesclomol for 24 h, the specific subpopulation with high JARID1B expression almost disappeared [27]. In addition, the combination of elesclomol with cisplatin also reversed the enrichment of high JARID1B cells caused by cisplatin monotherapy [27]. Compared to the control group, cisplatin treatment resulted in an abrupt increase in the high JARID1B expression subpopulation from 5 to 30%, while the combination of elesclomol dose-dependently reduced the percentage of this subpopulation [27]. This was also confirmed by Western blotting results, where the expression of JARID1B was downregulated in adherent cells and upregulated in dead cells in the supernatant after elesclomol treatment, indicating that elesclomol is somewhat selective for cells with a high expression of JARID1B. The dependence of this group of cells on oxidative ATP production may be the basis of their sensitivity to elesclomol [41].

The downregulation of thioredoxin-1 (TRX-1) expression and activation of mitochondrial respiration in cisplatin-resistant lung cancer cells have been found, the latter including increased cellular oxygen consumption and enhancement of mRNA of mitochondrial respiration-related enzymes such as argininosuccinate synthetase and fumarase [26]. In addition, cisplatin-resistant cells were more sensitive to elesclomol than parental cells were [26]. When TRX-1 expression was upregulated in cisplatin-resistant cells, the mitochondrial respiration intensity appeared to decrease, accompanied by the remission of cisplatin resistance [26]. However, more importantly, the sensitivity of cisplatin-resistant cells to elesclomol disappeared with the decrease in mitochondrial respiration intensity [26]. Thus, the sensitivity of cisplatin-resistant lung cancer cells to elesclomol is closely related to mitochondrial metabolism.

Elesclomol therapy increased ROS in cisplatin-resistant lung cancer cells [17, 26], a phenomenon partially reversed by the antioxidant N-acetylcysteine (NAC). The elesclomol treatment-induced intracellular ROS levels may be related to the decompensation of the intracellular antioxidant system. Oxidative damage caused by ROS ultimately leads to cell death [17].

#### Proteasome inhibitor-resistant cancer cells

On the one hand, massive protein synthesis is the basis for the exuberant proliferation of cancer cells [42]. On the other hand, a protein quality control system is crucial for cancer cells to relieve intracellular proteotoxic stress [42]. Proteasome inhibitors (PIs) are reasonable cancer therapeutic drugs that exhibit potent cancer suppression under *in vitro* culture conditions [42]. Unfortunately, tumors *in vivo* show strong adaptability to PI, which significantly limits the clinical application of PI [43].

Functional genomics analysis demonstrated that the generation of PI resistance is related to mitochondrial metabolism [28]. Mitochondrial OXPHOS was significantly enriched in PI-resistant cells [28]. Replacing glucose with galactose in culture medium resulted in an enhanced reliance on mitochondrial metabolism in cells [44]. The investigators screened 549 cancer cell lines for PI resistance in a mitochondrial metabolism-reliant state using PRISM, a barcoding method [28]. Despite the reduced viability of some cells due to galactose, the survival rate of cells in the mitochondrial metabolism-reliant state after PI treatment was significantly higher than that of the control group [28]. Subsequently, elesclomol emerged as one of the only three effective drugs in the screening of anticancer drugs for PI-resistant cells [28]. PI-resistant cells showed sensitivity to elesclomol, and elesclomol treatment in turn enhanced the sensitivity of PI-resistant cells to PI [28]. The results of subsequent animal experiments also validated the therapeutic effect of elesclomol on PI-resistant cancer cells [28]. Here, elesclomol was found to induce copper-dependent death, and the copper chelator tetrathiomolybdate (TTM), rather than the apoptosis inhibitor or ferroptosis inhibitor, reversed the cell death induced by elesclomol [28].

#### Molecularly targeted agent-resistant cancer cells

Vemurafenib is an anticancer agent targeting the BRAFV600E mutation in melanoma cells [45]. Its emergence has changed the clinical treatment paradigm for melanoma but is threatened by the rapid development of drug resistance in clinical practice [45].

Vemurafenib was found to significantly induce mitochondrial metabolism in BRAFV600E mutant melanoma cells, presenting as elevated basal and higher maximum respiratory capacity [20]. The investigators exposed melanocytes to different concentrations of vemurafenib for 2–3 months to obtain vemurafenib-resistant subcellular lines, and it was found that the vemurafenib-resistant cells also showed an elevation of mitochondrial metabolism, accompanied by the structural and functional complexity of mitochondria, which included an increase in the number of cristae in the mitochondria and an expansion of the cristae space [20]. Subsequently, it was found that vemurafenib-resistant cells exhibited a high degree of sensitivity to elesclomol compared to parental cells, consistent with its sensitivity to KCN [20], a mitochondrial complex IV inhibitor. These experiments demonstrated that vemurafenib-resistant cells are more sensitive to agents that target the key regulators of mitochondrial metabolism.

### Cancer cells with lower glycolytic activity are sensitive to elesclomol

Hypoxia is a characteristic of solid tumors. Tumors face the challenge of hypoxia in the process of either onset or metastasis [46]. Metabolic plasticity enables cancer cells to increase their reliance on anaerobic glycolytic metabolism in response to hypoxia [47]. Among many regulatory molecules, hypoxia-inducible factor-1α (HIF-1α) and its downstream protein pyruvate dehydrogenase kinase-3 (PDK3) are important factors in the metabolic plasticity induced by hypoxia [47].

Nucleus accumbens-1 is an upstream regulator of HIF-1α, and its induction of PDK3 enhances the glycolysis reliance of the ovarian cancer SKOV3 cell line and cervical cancer HeLa cell line [48]. After the silencing of intracellular nucleus accumbens-1 expression by exogenous plasmids, mitochondrial respiration was activated in both SKOV3 and HeLa cancer cells, which was more pronounced in hypoxia [48]. Meanwhile, nucleus accumbens-1 silencing enhanced the anticancer activity of elesclomol *in vitro* and *in vivo* [48].

Dichloroacetate (DCA) is a small molecule that targets PDK to shift cellular metabolism from glycolysis to mitochondrial metabolism [49]. Treatment with DCA is predicted to increase the reliance of cancer cells on mitochondrial metabolism. The combination of DCA with elesclomol has a synergistic inhibitory effect on melanoma cells cultured *in vitro* [25]. The combination also significantly retarded tumor growth in a preclinical model of human melanoma HBL cell line tumor grafts in mice [25].

### Cancer patients with low serum LDH levels are sensitive to elesclomol

LDH participates in the anaerobic glycolysis of cells, and its transcription is regulated by HIF-1α [50]. In cancer patients, elevated serum LDH levels are associated with poor prognosis [13] because serum LDH levels arise partly from cancer cells under hypoxia [13]. Hypoxia induces increased glycolytic metabolism in cancer cells, ultimately leading to cancer progression [51].

A phase III clinical trial of metastatic melanoma demonstrated that the serum LDH levels of patients correlate with sensitivity to elesclomol [12]. The purpose of this trial was to evaluate whether the addition of elesclomol to paclitaxel therapy prolongs PFS in patients with advanced melanoma [12]. The results showed that the addition of elesclomol was ineffective in patients with high serum LDH levels, but those patients with low serum LDH levels had a 1.6-month increase in median PFS [12]. The investigators speculate that this is because of the characteristics of high mitochondrial metabolism in patients with low serum LDH levels, which make them more sensitive to elesclomol [12]. Thus, serum LDH levels are expected to serve as a marker for whether patients are sensitive to elesclomol [12]. However, another study on acute myeloid leukemia suggested that hematologic tumors differed from solid tumors in that elevated LDH was not strongly associated with hypoxia [10]. In clinical trials of elesclomol therapy, serum LDH levels should not be applied for screening patients with hematologic tumors [10].

## The anticancer mechanism of elesclomol

**Fig. 2:**
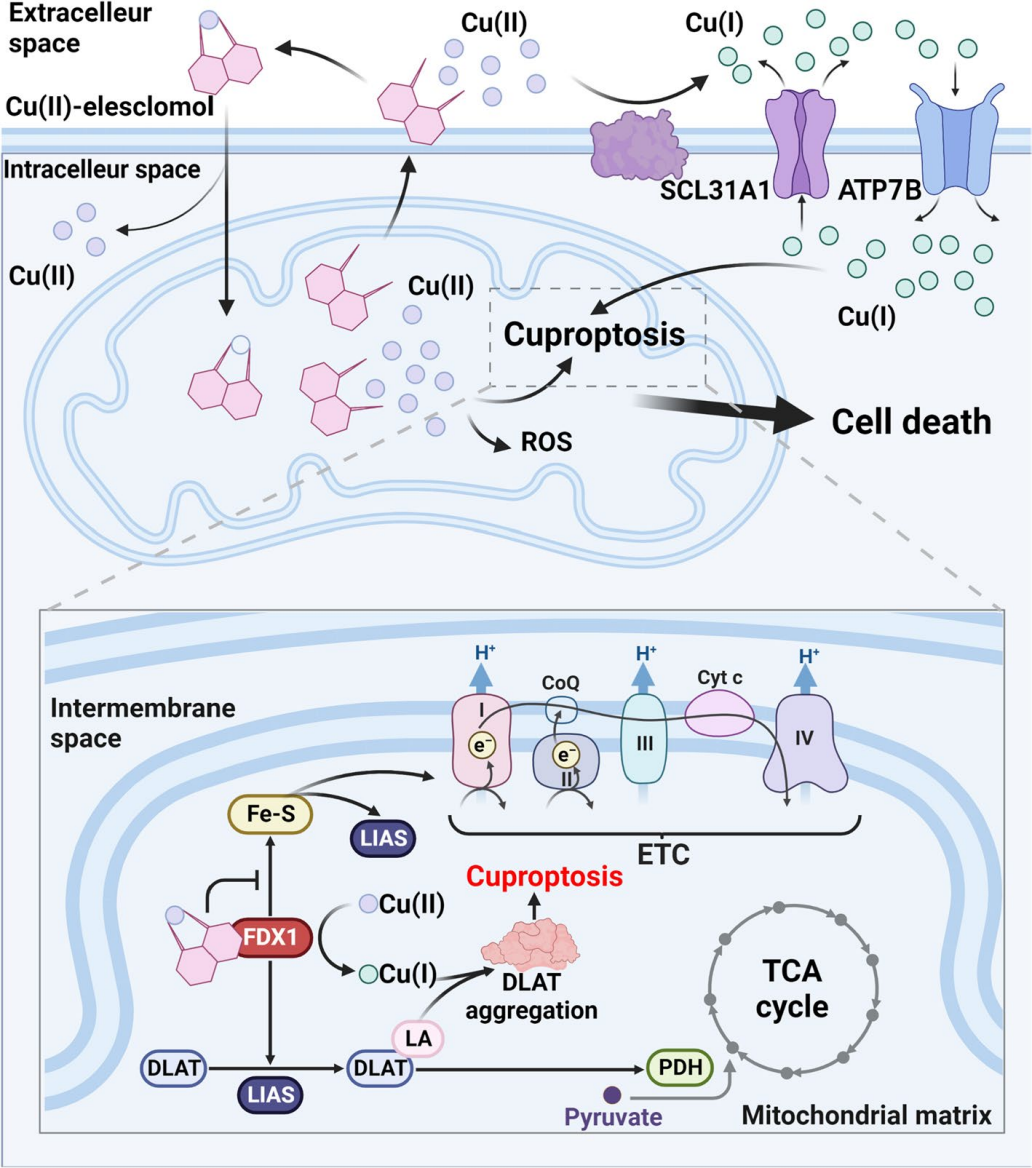
Schematic diagram of the mechanism of elesclomol-induced cell death. Elesclomol shuttles inside and outside the cell to selectively transport extracellular Cu(II) to mitochondria, where Cu(II) accumulated in mitochondria induces ROS production and triggers cuproptosis. FDX1, a critical enzyme in the occurrence of pyroptosis, reduces Cu(II) to Cu(I) in mitochondria while promoting the lipoylation of DLAT, an enzyme participating in the formation of the PDH complex and affecting the mitochondrial TCA cycle. The reduced Cu(I) binds to lipoylated DLAT to promote its oligomerization, ultimately leading to the occurrence of cuproptosis. FDX1 also promotes Fe-S synthesis, while Cu(I) inhibits this process. Fe-S is an essential component of LIAS and ETC, the key enzymes in DLAT lipoylation, but its relationship with cuproptosis is unclear. In addition, the regulation of intracellular Cu(I) levels by membrane copper ionophores, such as SCL31A1 and ATP7B, is also associated with the occurrence of cuproptosis. DLAT: dihydrolipoamide S-acetyltransferase; ETC: electron transfer chain; FDX1: ferredoxin 1; Fe-S: iron-sulfur proteins; LA: lipoamide; LIAS: lipoyl synthase; PDH: pyruvate dehydrogenase; ROS: reactive oxygen species; TCA: tricarboxylic acid cycle

There is no definite conclusion on the cancer-suppressive mechanism of elesclomol. The mainstream view is that elesclomol is an oxidative stress inducer. Excessive ROS in cells may induce cell apoptosis [16, 22]. However, some studies have also found that cellular damage induced by elesclomol also involves DNA damage and cell cycle arrest [52]. In addition, it was reported that elesclomol induces ferroptosis [53]. Recently, elesclomol has also been found to be a cuproptosis inducer [7]. Cuproptosis has just been discovered as a new form of cell death [7]. We believe that the induction of cuproptosis may be a novel and reasonable explanation for the anticancer mechanism of elesclomol (Fig. 2).

### The anticancer effect of elesclomol is heavily reliant on copper ions

Although the anticancer mechanism of elesclomol is still controversial, there is no doubt that the anticancer effect of elesclomol depends on the existence of copper ions in the external cellular environment [6, 7].

Extracellularly, elesclomol can form a stable 1:1 complex with Cu(II) [54]. Afterward, elesclomol shuttles back and forth between the inside and outside of the cell to transport copper ions into the cell [6]. Notably, unlike other copper ionophores, such as DSF, cellular copper levels are selectively enriched in mitochondria after elesclomol treatment. Elesclomol induces a much higher rise in intracellular copper ions than DSF at the same concentration [6]. Meanwhile, the use of elesclomol has been reported to degrade copper-transporting ATPase 1 (ATP7A) in colon cancer cells, a protein that mediates intracellular copper export [55]. The degradation of ATP7A by elesclomol further leads to the enrichment of copper ions in the mitochondria of cancer cells [53].

The toxicity of elesclomol originates from the enrichment of copper ions in mitochondria, not from the complex of elesclomol with Cu(II) or elesclomol itself [6]. As early as 2012, researchers found that the killing effect of elesclomol on MDA-MB435 melanoma cells was completely lost when cells were cultured without serum, which was the only source of copper in the culture medium [6]. The addition of copper to a serum-free medium rescued the anticancer effect of elesclomol, whereas iron, manganese, and zinc did not contribute to the rescue of elesclomol’s effect [6]. A recent report on cuproptosis has obtained consistent experimental results in monocytes and lung cancer NCIH2030 cells [7]. In addition, some researchers paired elesclomol with redox inert metals Ni(II) and Pt(II) [56]. They found that compared to Cu(II)-elesclomol, the killing effect of Ni(II)-elesclomol and Pt(II)-elesclomol on K562 leukemia cells decreased 34 times and 1040 times, respectively [56]. Therefore, the toxicity of elesclomol to cancer cells is exerted mainly through copper ions.

### The old view—induction of oxidative stress for cancer suppression

Elesclomol was found to enhance oxidative stress and induce ROS production in various cells, including melanoma cells [6, 16, 18–20, 23], lung cancer cells [17, 22], GSCs [24], and 14 types of gynecological tumor cells [21]. Among them, melanoma cells have spontaneous enhancement of mitochondrial metabolism [18]; lung cancer cells and melanoma cells enhance mitochondrial metabolism in the process of drug resistance [17, 20]; and GSCs heavily reliant on mitochondrial metabolism [24]. ROS is an inevitable side-product of redox reactions. The aerobic characteristics of mitochondrial metabolism make it a major source of intracellular ROS [57]. The enhanced mitochondrial metabolism in these cells undoubtedly leads to increased ROS production, which is further strengthened by using elesclomol, ultimately leading to decompensation of the intracellular antioxidant system. The induction of intracellular ROS by elesclomol may account for the sensitivity of these cells to elesclomol.

In addition, elesclomol has a certain effect on the intracellular antioxidant system. Elesclomol has been reported to downregulate TRX in cisplatin-resistant lung cancer cells [17]. TRX is a key molecule in maintaining the intracellular reduction reaction [58]. Moreover, elesclomol also downregulates glutathione (GSH) in cisplatin-resistant lung cancer cells [17]. GSH is an essential member of the intracellular antioxidant system that facilitates the clearance of intracellular ROS [59]. Elesclomol not only induces the production of intracellular ROS but also impedes the clearance of ROS.

The induction of ROS by elesclomol seems uncontroversial, but is ROS production necessary for elesclomol to exert cytotoxicity? Several studies have attempted to reverse the induction of ROS by elesclomol using the ROS scavenger N-acetylcysteine (NAC); only in some cells did the use of NAC reverse ROS production [16, 60]. The effect of NAC on the toxic effects of elesclomol is controversial. On the one hand, studies have shown that the use of 0.1 mM and 10 mM NAC can reverse the cytotoxic effects of elesclomol on multiple small cell lung cancer (SCLC) cell lines, including SCLC 1, SCLC SR2, SCLC B, and SCLC BC [17] and non-small cell lung cancer cells A549 [22]. On the other hand, in GSCs, 5 mM NAC did not help to reverse the cytotoxicity of elesclomol, while 10 mM NAC had only a partial reversal effect [24]. Another study also showed that 5 mM NAC only slightly alleviated the loss of activity of three cell lines, NCIH2030, A549, and HCC4009, in response to elesclomol [7]. Thus, the anticancer effect of elesclomol is partly related to its induction of ROS, but there should be a more critical mechanism to explain the cancer cytotoxicity of elesclomol.

### A new view—induction of cuproptosis for cancer suppression

The latest view is that elesclomol can also induce a specific copper-dependent cell death, namely, cuproptosis [7]. None of the currently known inhibitors of other forms of cell death can reverse elesclomol-induced cell death, including the apoptosis inhibitors Z-VAD-FMK and Boc-D-FMK [7, 24], the ferroptosis inhibitor ferrostatin-1 [7, 24], the necrotrophic apoptosis inhibitor necrostatin-1 [7, 24] and the autophagy inhibitor 3-methyladenine [24].

Elesclomol-induced cuproptosis is associated with mitochondrial metabolism [7]. Cells that primarily relied on mitochondrial metabolism for energy production were 1000 times more sensitive to elesclomol than cells that primarily relied on anaerobic glycolysis [7]. At the same time, metabolomic analysis revealed that ABC1, an elesclomol-sensitive human lung cancer adenocarcinoma cell, exhibited dysregulation of TCA-related metabolites after elesclomol treatment, which included the upregulation of glutamine, α-ketoglutarate (α-KG), succinate, citric acid, cis-aconitate, and sedoheptulose 7-phosphate [7]. In contrast, there was no change in TCA metabolites in elesclomol-resistant lung cancer cells A549 [7], suggesting that the occurrence of elesclomol-induced cuproptosis is associated with the TCA cycle. In addition, the mitochondrial stress test showed that elesclomol treatment had no effect on basal respiration but significantly inhibited maximal respiration in ABC1 cells [7]. Additionally, the use of elesclomol did not affect the production of ATP [7], which is consistent with the finding that the mitochondrial OXPHOS uncoupler carbonyl cyanide-p-trifluoromethoxyphenylhydrazone (FCCP) did not affect the cytotoxicity of elesclomol [7]. Therefore, the authors speculate that elesclomol does not directly inhibit the electron transport chain.

Elesclomol-induced cuproptosis mainly depends on the oligomerization of lipoylated dihydrolipoamide S-acetyltransferase (DLAT) [7]. The occurrence of cuproptosis is dependent on FDX1. Elesclomol specifically transfers extracellular Cu(II) to mitochondria, after which FDX1 reduces Cu(II) to Cu(I), a more toxic form of copper ion [7]. Additionally, FDX1 is an upstream regulator of mitochondrial protein lipoylation [7]. Protein lipoylation is a highly conserved posttranslational modification process of lysine [61]. As an essential component subunit of the pyruvate dehydrogenase (PDH) complex, lipoylation of DLAT is required for the PDH complex to regulate pyruvate entry into the TCA cycle [61]. The enrichment of Cu(I) in mitochondria caused by elesclomol and FDX1 ultimately leads to oligomerization of DLAT in mitochondria, which depends on the direct binding of Cu(I) to lipoylated DLAT [7].

Elesclomol treatment also results in the loss of iron-sulfur (Fe-S) cluster proteins in mitochondria [7, 28]. Fe-S cluster protein synthesis requires FDX1 as a reducing agent [28], and the specific binding of Cu(II)-elesclomol to FDX1 inhibits the synthesis process [28]. Fe-S cluster proteins are involved in the synthesis of ETC in mitochondria; whereas, elesclomol-induced cuproptosis and ETC do not seem to be directly related [7]. The role of Fe-S cluster proteins in elesclomol-induced cuproptosis remains to be further investigated.

## Clinical application prospects of elesclomol targeting mitochondrial metabolism in cancer therapy

### Elesclomol treatment for cancers with high mitochondrial metabolism

In some types of cancer, the enhancement of mitochondrial metabolism is spontaneous, which may be driven by genetic mutations. Meanwhile, enhanced mitochondrial metabolism is only shown in some subtypes in other types of cancer [3].

**Fig. 3:**
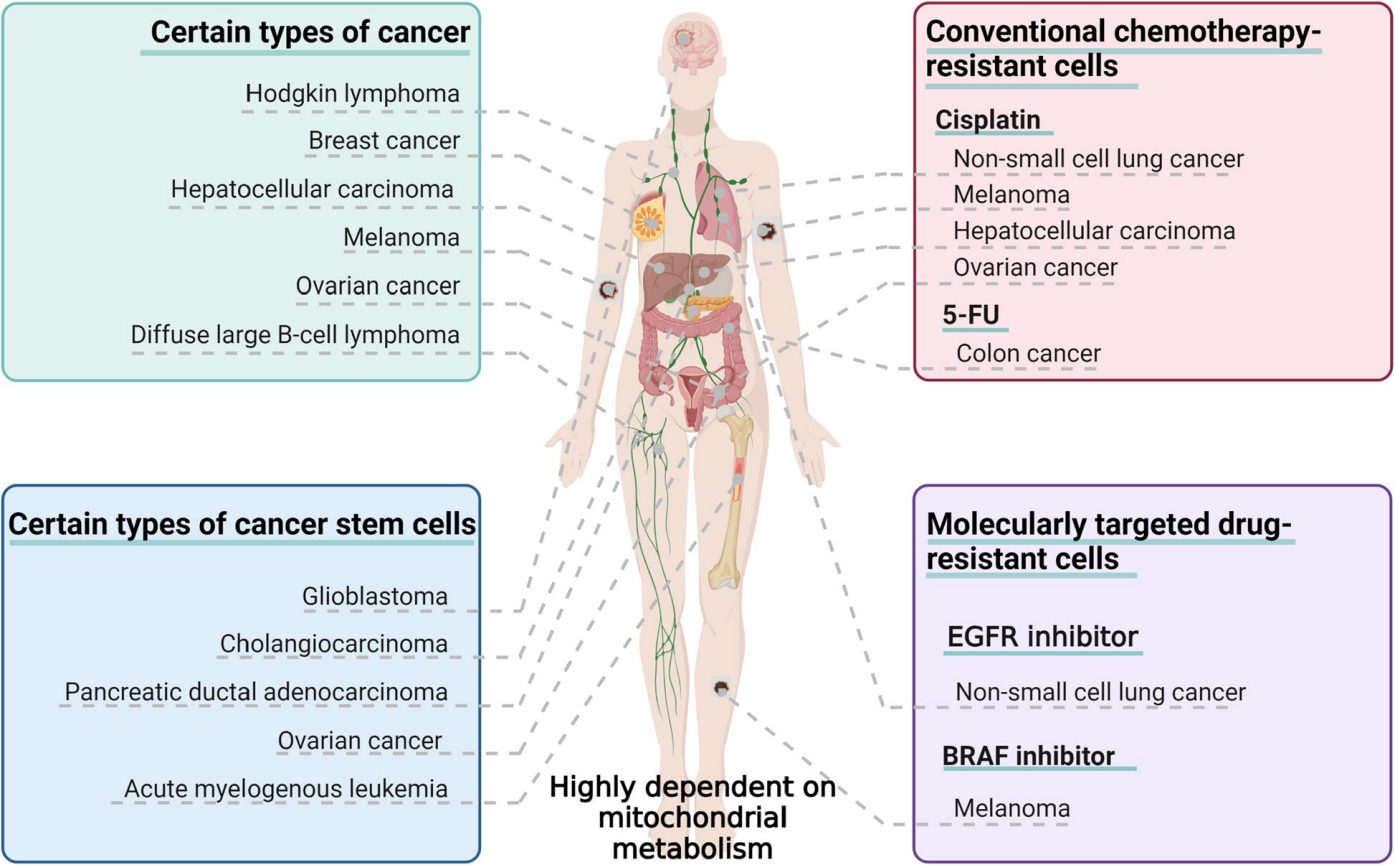
Cancers highly dependent on mitochondrial metabolism. Cancers such as melanoma, breast cancer, and ovarian cancer show spontaneous enhancement of mitochondrial metabolism. Cancer stem cells of glioblastoma, ovarian cancer, cholangiocarcinoma, and other cancers highly depend on mitochondrial metabolism. Increased dependence on mitochondrial metabolism is seen in drug-resistant cancer cells generated in some anticancer treatments, including cisplatin-resistant melanoma and hepatocellular carcinoma from conventional chemotherapies and 5-FU-resistant colon cancer, BRAF inhibitor-resistant melanoma from molecularly targeted drugs, and EGFR inhibitor-resistant non-small cell lung cancer. 5FU: 5-Fluorouracil; BRAF: v-Raf murine sarcoma viral oncogene homolog B1; EGFR: epidermal growth factor receptor

Melanoma [18, 62], breast cancer [63], Hodgkin's lymphoma [64], and hepatocellular carcinoma cells [65] show a marked increase in mitochondrial metabolism, whereas high-grade serous ovarian cancer [66] and diffuse large B-cell lymphoma [67] show metabolic heterogeneity; that is, enhanced mitochondrial metabolism is only observed in some subsets. In addition, cancer stem cells from solid tumors, such as pancreatic ductal adenocarcinoma [68], glioblastoma [34], ovarian cancer [69] and cholangiocarcinoma [70], as well as stem cells of acute myeloid leukemia [71], also show a heavy reliance on mitochondrial metabolism (Fig. 3).

Based on the specific relationship between elesclomol and cancer metabolism, selecting cancers with high mitochondrial metabolism for clinical trials seems more likely to yield desirable results. Hypoxia leads to tumor progression and induces glycolytic metabolism, which reduces the sensitivity to elesclomol. With this understanding, hypoxic biological markers can be used to screen for patients sensitive to elesclomol. For example, LDH is a biomarker of tumor hypoxia [13], and patients with low LDH levels are more sensitive to elesclomol [12]. Genomic studies help identify new tumor hypoxia biomarkers [72] and hypoxia-related genes [73]. It is important to screen specific populations for elesclomol treatment with the guidance of genomics and to carry out precise therapy.

Cancer stem cells play a crucial role in cancer development, drug resistance, and recurrence. They offer self-renewal properties to cancer and facilitate long-term cancer survival [74, 75]. Due to the stronger reliance of cancer stem cells on mitochondrial metabolism, elesclomol therapy may reduce the ability of cancer cells to adapt to the harsh environment, delay recurrence, and improve the survival of patients.

### Elesclomol treatment for drug-resistant cancer cells

Studies have found that cancer cells resistant to anticancer drugs, including conventional chemotherapeutic drugs [76, 77] and molecularly targeted drugs [40, 78], are associated with metabolic reprogramming.

Elesclomol has been found to have a potent killing effect on cisplatin-resistant lung cancer cells [17, 26] and melanoma cells [27], vemurafenib-resistant melanoma cells [20], and PI inhibitor-resistant breast cancer cells [28], which is associated with upregulation of mitochondrial metabolism in these cells. In addition, mitochondrial metabolism was also enhanced in 5-fluorouracil-resistant colon cancer cells [76] and cisplatin-resistant hepatocellular carcinoma [79], ovarian cancer cells [77]. The epidermal growth factor receptor (EGFR) inhibitor erlotinib, a key drug in the clinical treatment of small cell lung cancer [80], was found to have an off-target effect in activating mitochondrial metabolic function in non-small cell lung cancer [78]. It is of great clinical significance to study the inhibitory effect of elesclomol on these drug-resistant cells that show changes in mitochondrial function.

Furthermore, using elesclomol as an adjuvant in chemotherapy in combination with other drugs seems to be a viable strategy. Treatment with chemotherapeutic drugs forces cancer cells to selectively alter their metabolic patterns, increasing their reliance on mitochondrial metabolism to adapt to the changes brought about by the drugs. However, cells with high mitochondrial metabolism are sensitive to elesclomol [16]. Under the dual stress of both chemotherapeutic drugs and elesclomol, cancer cells face opposite pressure, which may help prevent the development of drug resistance in cancer cells.

### Combination of elesclomol with glycolysis inhibitors for cancer therapy

Glycolysis has been proposed as a target for cancer therapy for many years [81]. A number of small molecules with glycolysis inhibitory activity have shown satisfactory anticancer activity *in vivo* and *in vitro*. For instance, 2-Deoxy-d-glucose (2-DG) is a compound that blocks glycolysis by competitively inhibiting the rate-limiting enzyme hexokinase intracellularly and leading to inhibition of glycolysis [82]. 2-DG is routinely used as a radioactive tracer in clinical practice and has been found to be safe [82]. Based on its glycolytic inhibitory effect, the role of 2-DG in cancer therapy is gaining attention [82]. Recently, glycolysis inhibitors such as 6-aminonicotinamide [83] and 3-bromopyruvic [84] have gradually shown new therapeutic value in cancer treatment. Several clinical trials have also been carried out [81].

The combination of the above glycolytic inhibitors with elesclomol may be an effective solution. Although there are no reports on the combination of elesclomol and glycolysis inhibitors in the treatment of cancer, the related investigation predicts that the combination of these two will achieve good therapeutic effects. In preclinical models, promising results have been achieved with the combination of elesclomol and the PDK inhibitor DCA, a small molecule that shift cellular metabolism from glycolysis to mitochondrial metabolism [25]. Cancer metabolic plasticity enables cells to increase their reliance on mitochondrial metabolism in the face of glycolytic inhibition, which facilitates the function of elesclomol.

### Safety of elesclomol in cancer therapy

The safety of drugs is also an issue worthy of further attention. Some studies have reported that elesclomol's cytotoxicity seems to be selective for cancer cells [6]. Human peripheral blood mononuclear cells (PMBCs) were unaffected at concentrations that had a significant killing effect on cancer cells, and elesclomol could not induce copper ion enrichment in PMBCs [6]. However, some studies have reported that elesclomol has certain effects on mitochondrial function in normal mammalian cells, and the treatment of CV-1 cells with more than 40 μM elesclomol increases the production of ROS in mitochondria while significantly reducing the mitochondrial membrane potential [85].

According to existing reports, nearly one thousand patients have received high doses of elesclomol in clinical trials. Good tolerance of elesclomol by patients is a common feature of these trials. For example, the maximum tolerated dose of elesclomol was up to 438 mg/m^2^ in patients with solid tumors in a phase I trial [9]. In past clinical trials, no patients have been reported to develop elesclomol-related organic or functional impairment [5, 9–11]. Therefore, elesclomol treatment has a high safety profile.

## Conclusion

Elesclomol targets mitochondrial metabolism, and its induction of cuproptosis is a crucial discovery in cancer research. Cuproptosis, a novel form of cell death, has made an essential supplement to the specific anticancer mechanism of elesclomol. In turn, further understanding the mechanism of its cancer suppression will help in understanding the exact process of cuproptosis.

Cancer cells that heavily rely on mitochondrial metabolism are extremely sensitive to elesclomol [7]. Existing studies have reported significant inhibitory effects of elesclomol on a variety of cells, including cancer stem cells [24], drug-resistant cells [27], and cells with lower glycolytic activity [25], due to enhanced mitochondrial metabolism. In addition, many clinical trials have been conducted to ensure the safety of elesclomol in clinical application. Serum LDH levels may be a potential biomarker for assessing patients’ sensitivity to elesclomol [12].

Based on the characteristics of cancer cells in which those with high mitochondrial metabolism are much more sensitive to elesclomol, further research is suggested to identify cancer types or subtypes sensitive to elesclomol, to validate the efficacy of elesclomol combined with chemotherapeutic drugs or glycolysis inhibitors in preclinical models and to seek the clinical application value of such combinations. In clinical trials, screening populations susceptible to elesclomol based on serum LDH levels or other biological markers of hypoxia would be more conducive to obtaining desirable trial results.

## Data Availability

Not applicable.

## Abbreviations

ATP: Adenosine triphosphate
ALDH: Aldehyde dehydrogenase
FCCP: Carbonyl cyanide-p-trifluoromethoxyphenylhydrazone
DCA: Dichloroacetate
DLAT: Dihydrolipoamide S-acetyltransferase
DSF: Disulfiram
ETC: Electron transport chain
EGFR: Epidermal growth factor receptor
GSCs: Glioblastoma stem cells
GSH: Glutathione
HIF-1α: Hypoxia-inducible factor-1α
Fe-S: Iron-sulfur proteins
JARID1B: Jumonji AT-rich interactive domain 1B
LDH: Lactate dehydrogenase
NAC: N-Acetyl-L-cysteine
OC: Ovarian cancer
OXPHOS: Oxidative phosphorylation
PMBCs: Peripheral blood mononuclear cells
PFS: Progression-free survival
PI: Proteasome inhibitorprotease inhibitor
PDH: Pyruvate dehydrogenase
PDK3: Pyruvate dehydrogenase kinase-3
ROS: Reactive oxygen species
SCLC: Small cell lung cancer
TTM: Tetrathiomolybdate
TRX-1: Thioredoxin-1
TCA: Tricarboxylic acid cycle
TICs: Tumor-initiating cells
α-KG: α-Ketoglutarate
2-DG: 2-Deoxy-d-glucose
